# Respiratory motion-compensated high-resolution 3D whole-heart T1ρ mapping

**DOI:** 10.1186/s12968-020-0597-5

**Published:** 2020-02-03

**Authors:** Haikun Qi, Aurelien Bustin, Thomas Kuestner, Reza Hajhosseiny, Gastao Cruz, Karl Kunze, Radhouene Neji, René M. Botnar, Claudia Prieto

**Affiliations:** 1 School of Biomedical Engineering and Imaging Sciences, King’s College London, 3rd Floor, Lambeth Wing, St Thomas’ Hospital, Lambeth Palace Rd, London, SE1 7EH UK; 2 Siemens Healthcare, MR Research Collaborations, Frimley, UK; 3 Escuela de Ingeniería, Pontificia Universidad Católica de Chile, Santiago, Chile

**Keywords:** T1ρ mapping, T1ρ preparation, Free-breathing, Myocardial tissue characterization, Non-contrast, Cardiovascular magnetic resonance

## Abstract

**Background:**

Cardiovascular magnetic resonance (CMR) T1ρ mapping can be used to detect ischemic or non-ischemic cardiomyopathy without the need of exogenous contrast agents. Current 2D myocardial T1ρ mapping requires multiple breath-holds and provides limited coverage. Respiratory gating by diaphragmatic navigation has recently been exploited to enable free-breathing 3D T1ρ mapping, which, however, has low acquisition efficiency and may result in unpredictable and long scan times. This study aims to develop a fast respiratory motion-compensated 3D whole-heart myocardial T1ρ mapping technique with high spatial resolution and predictable scan time.

**Methods:**

The proposed electrocardiogram (ECG)-triggered T1ρ mapping sequence is performed under free-breathing using an undersampled variable-density 3D Cartesian sampling with spiral-like order. Preparation pulses with different T1ρ spin-lock times are employed to acquire multiple T1ρ-weighted images. A saturation prepulse is played at the start of each heartbeat to reset the magnetization before T1ρ preparation. Image navigators are employed to enable beat-to-beat 2D translational respiratory motion correction of the heart for each T1ρ-weighted dataset, after which, 3D translational registration is performed to align all T1ρ-weighted volumes. Undersampled reconstruction is performed using a multi-contrast 3D patch-based low-rank algorithm. The accuracy of the proposed technique was tested in phantoms and in vivo in 11 healthy subjects in comparison with 2D T1ρ mapping. The feasibility of the proposed technique was further investigated in 3 patients with suspected cardiovascular disease. Breath-hold late-gadolinium enhanced (LGE) images were acquired in patients as reference for scar detection.

**Results:**

Phantoms results revealed that the proposed technique provided accurate T1ρ values over a wide range of simulated heart rates in comparison to a 2D T1ρ mapping reference. Homogeneous 3D T1ρ maps were obtained for healthy subjects, with septal T1ρ of 58.0 ± 4.1 ms which was comparable to 2D breath-hold measurements (57.6 ± 4.7 ms, *P* = 0.83). Myocardial scar was detected in 1 of the 3 patients, and increased T1ρ values (87.4 ± 5.7 ms) were observed in the infarcted region.

**Conclusions:**

An accelerated free-breathing 3D whole-heart T1ρ mapping technique was developed with high respiratory scan efficiency and near-isotropic spatial resolution (1.7 × 1.7 × 2 mm^3^) in a clinically feasible scan time of ~ 6 mins. Preliminary patient results suggest that the proposed technique may find applications in non-contrast myocardial tissue characterization.

## Background

Myocardial infarction detection is important for the evaluation of post-infarction left ventricular remodelling [1]. Late gadolinium enhanced (LGE) cardiovascular magnetic resonance (CMR) imaging is widely used to detect scar, because of its high contrast between the infarcted region and the surrounding myocardium [2, 3]. However, LGE imaging requires the administration of gadolinium-based contrast agents, which may be contraindicated in patients with severe renal impairment, due to poor renal clearance of the contrast agent [4]. Furthermore, there has been increasing concern about the contrast agent deposition in the brain [5]. Endogenous contrast imaging techniques, such as T1ρ CMR, have been proposed to address this problem [6–8].

T1ρ, the T1 relaxation time in the rotating frame, is a sensitive marker for assessment of macromolecular-water interaction, and has shown potential for diagnostic imaging of cartilage [9, 10], liver cirrhosis [11, 12] and acute cerebral infarction [13]. Promising results of T1ρ CMR were also observed for acute and chronic myocardial infarction characterization [7, 14] without the need of exogenous contrast agents. A study of end-stage renal disease patients showed that T1ρ can better characterize myocardial injury than native T1 and T2 maps [6]. However, current 2D myocardial T1ρ mapping requires multiple breath-holds to acquire several T1ρ-weighted images for pixel-wise T1ρ fitting [6, 15], which results in limited coverage and spatial resolution. Furthermore, the different breath-hold positions may lead to misregistration artifacts and inaccuracy of T1ρ estimation [15]. Respiratory gating can be used as an alternative to limit respiratory motion during free-breathing acquisitions. By using respiratory diaphragmatic navigator gating, a 3D free-breathing T1ρ mapping technique has recently been proposed to achieve whole-heart coverage [16]. This technique employed compressed sensing reconstruction with spatial total variation to accelerate the acquisition. However, due to the reduced acquisition efficiency by respiratory gating and the need of recovery heartbeats between T1ρ preparations, long scan times are required for the sequence with compromised spatial resolution (1.9 × 1.9 × 6 mm^3^, scan time ~ 18 min) [16].

In this study, we propose a free-breathing motion-compensated 3D whole heart T1ρ mapping technique with near-isotropic spatial resolution (1.7 × 1.7 × 2 mm^3^) and predictable and clinically feasible scan time (~ 6 min). The proposed electrocardiogram (ECG)-triggered sequence acquires different T1ρ-weighted volumes sequentially with increasing T1ρ spin-lock times. Similar to a previous cardiac T2 mapping technique [17], a saturation pulse is adopted to reset the magnetization in each heartbeat, reducing the influence of heart rate variability during acquisition and removing the requirement of recovery heartbeats between T1ρ preparations. The acquisition is accelerated using an undersampled variable-density 3D Cartesian sampling with spiral-like order (VD-CASPR) [18] and respiratory motion compensation is performed based on 2D image-navigators, enabling 100% respiratory scan efficiency (i.e. no respiratory gating) [19]. Motion-corrected undersampled k-space data is reconstructed using a multi-contrast 3D patch-based low-rank algorithm (HD-PROST) [20].

The accuracy of the proposed technique was investigated in phantom experiments. 3D whole heart T1ρ maps were demonstrated in healthy subjects and patients with myocardial infarction.

## Methods

### Pulse sequence

**Fig. 1:**
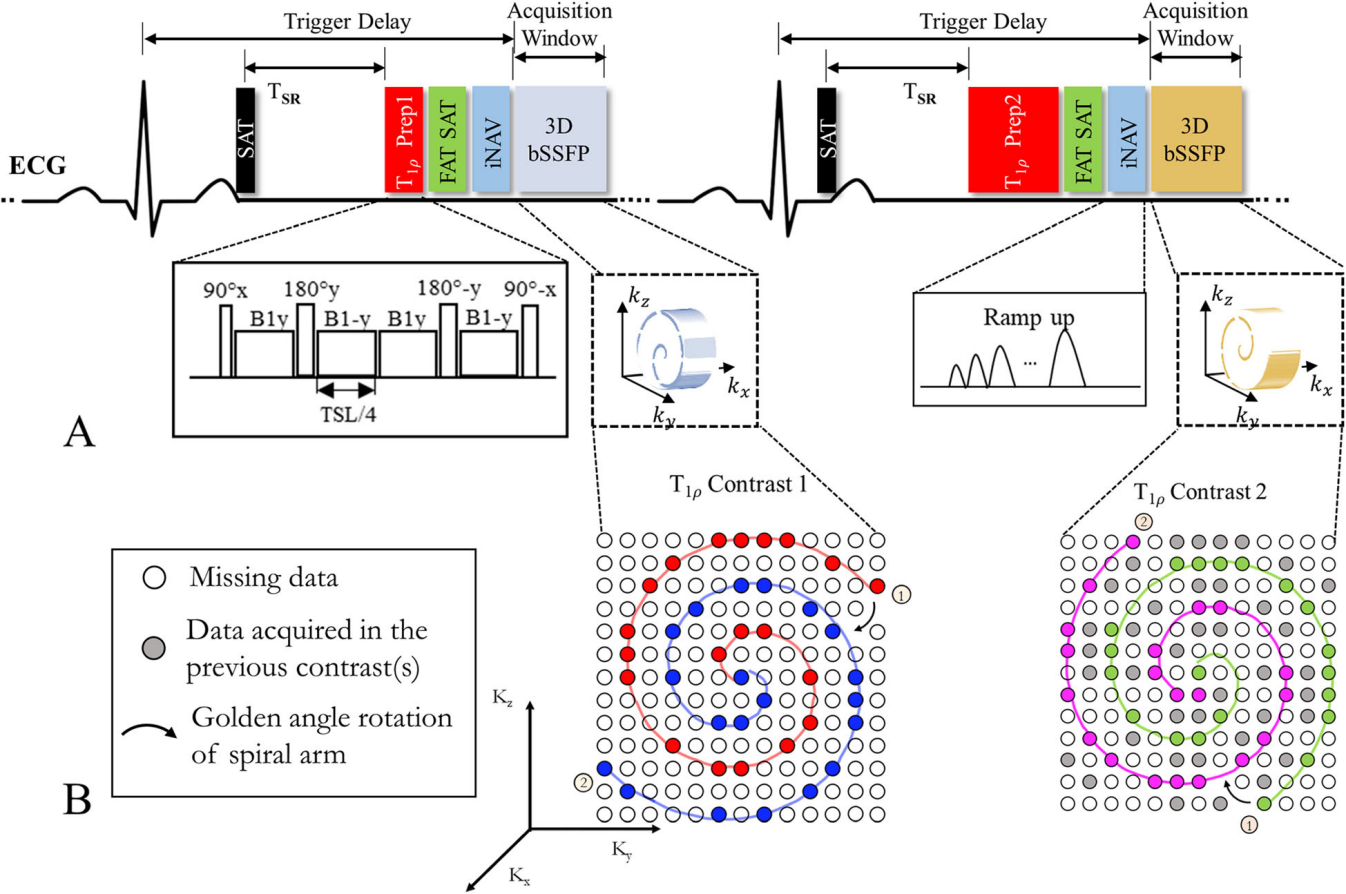
Sequence diagram for the proposed free-breathing electrocardiogram (ECG)-triggered 3D T1ρ mapping acquisition. Acquisitions of two different T1ρ contrasts with 3D balanced steady-state free precession (bSSFP) readouts are shown. A saturation pulse (SAT) is used to reset the magnetization in each heartbeat. After a constant delay (T_SR_), T1ρ preparation (T1ρ prep) is performed followed by fat saturation pulse (FAT SAT), whereas 2D image navigators (iNAV) are acquired using the ramp-up preparation pulses of the bSSFP (**a**). The trigger delay is kept constant for all T1ρ preps (with different durations) by adjusting the time between ECG and SAT. An undersampled variable-density 3D Cartesian trajectory with spiral-like order is used for acceleration, spiral-like arms are rotated with golden angle step within and between T1ρ contrasts (**b**)

The proposed 3D T1ρ mapping sequence is performed under free-breathing with mid-diastolic ECG-triggering and consists of a non-selective saturation pulse (SAT), T1ρ preparation (T1ρ prep), fat suppression using spectral pre-saturation with inversion recovery, 2D image based navigator (iNAV) [19] and 3D balanced steady-state free-precession (bSSFP) readout (Fig. 1a). Five differently T1ρ-weighted volumes are acquired sequentially. A 3D variable-density Cartesian sampling with spiral-like profile order (VD-CASPR) [18] with an undersampling factor of 3.8 is employed. One spiral-like arm is acquired per cardiac cycle, and spiral-like arms are rotated with a golden-angle order within and between T1ρ contrasts (Fig. 1b) to ensure incoherent undersampling artifacts along the spatial and contrast dimensions.

In the T1ρ preparation, besides the tip-down, tip-up pulses (90_±*x*_) and four separate spin-lock pulses with alternating phases (*B*1_±*y*_), 2 refocusing pulses with opposite phases (180_±*y*_) are added to make the T1ρ prep more robust to both B1 and B0 inhomogeneities [21, 22], resulting in the cluster (90_*x*_ − *B*1_*y*_ − 180_*y*_ − *B*1_−*y*_ − *B*1_*y*_ − 180_−*y*_ − *B*1_−*y*_ − 90_−*x*_) shown in Fig. 1a. The B1 amplitude of spin-lock pulse is set to 400 Hz. Five different T1ρ-weightings were acquired with T1ρ preps of five increasing spin-lock times (TSL = 0, 10, 20, 35, 50 ms). A volume without T1ρ prep (TSL = 0), which has the highest signal-to-noise ratio (SNR), is included to increase the T1ρ decay range. For TSL = 0, the tip-down, tip-up pulses and a crusher gradient are still performed to ensure more consistent influence of radiofrequency flip angle imperfections on all T1ρ-weightings.

Previous myocardial T1ρ mapping techniques acquire data in every other heartbeat to allow magnetization recovery [15, 16]. However, the idle cardiac cycles lead to increased scan time. Moreover, heart rate variability during the scan results in variable recovery times and can influence the T1ρ estimation accuracy [16]. In the proposed sequence, SAT is applied after the ECG trigger with a fixed delay (T_SR_) to the start of T1ρ prep, aiming to null the magnetization history at the start of each heartbeat and minimise heart rate dependency during the scan [17]. This enables data acquisition in every heartbeat without the need of recovery heartbeats. To ensure the same magnetization before each T1ρ prep and the same cardiac motion state for the acquisition in each cardiac cycle, T_SR_ and trigger delay (the time between the trigger and acquisition, Fig. 1a) should be fixed for all T1ρ preparations with different TSLs. In the cardiac cycle where T1ρ preparation is performed with the longest TSL, the time between the ECG trigger and SAT is made as short as possible for maximal saturation recovery. For T1ρ prep with shorter TSL, a delay time is inserted between the ECG trigger and SAT to ensure the same T_SR_ and trigger delay for all T1ρ preps.

**Fig. 2:**
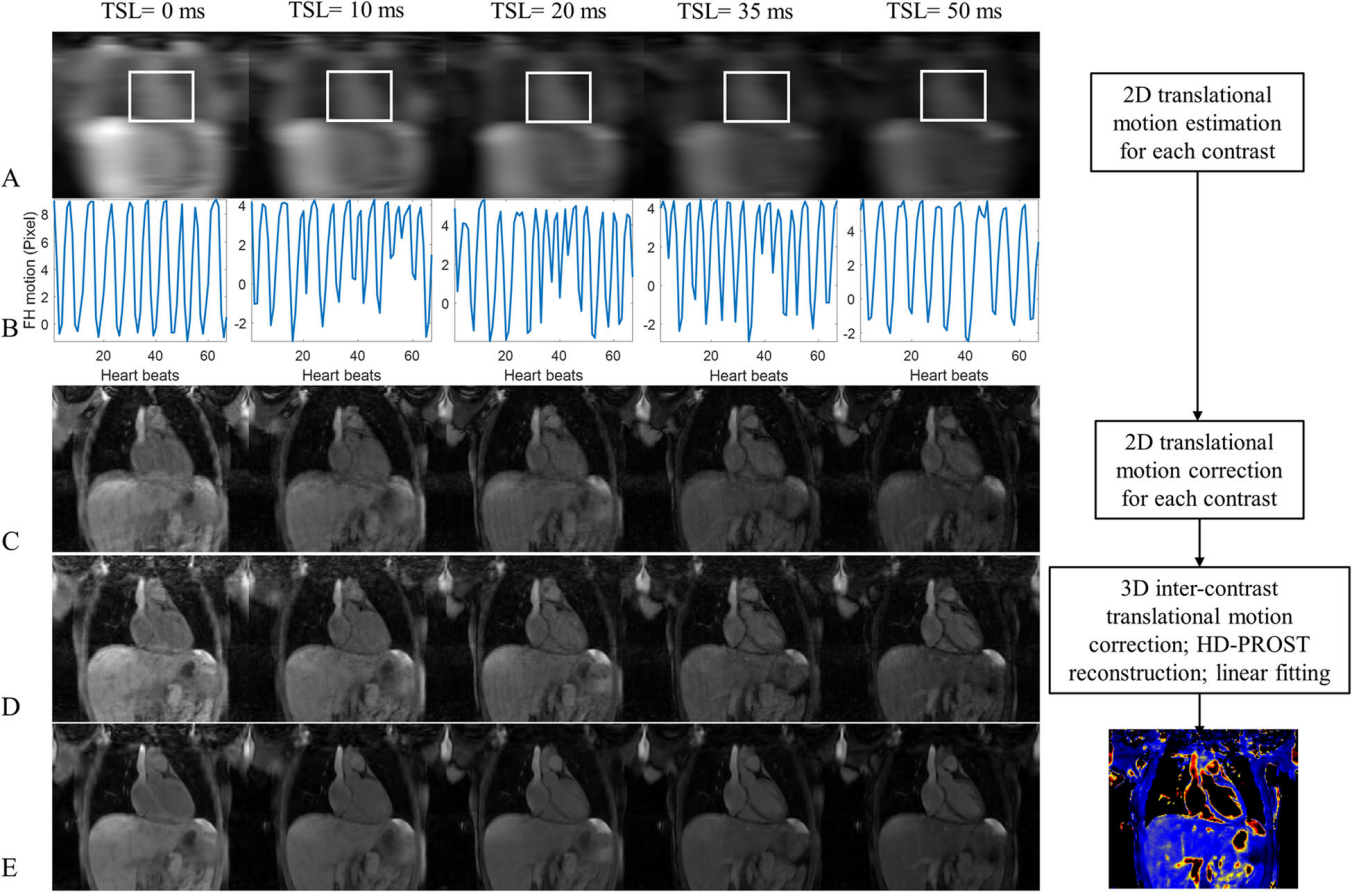
**a** The 2D image navigators (iNAVs) acquired with T1ρ preparations of five different spin-lock times (TSL). **b** The measured foot-head (FH) motion curves from iNAV of each contrast. **c** The motion-corrupted undersampled multi-contrast T1ρ-weighted images. **d** 2D translational motion-corrected undersampled multi-contrast T1ρ-weighted images. **e** 3D inter-contrast translational motion corrected and HD-PROST reconstructed images. T1ρ maps were obtained by pixel-wise linear fitting of the multi-contrast images in (**e**)

The 2D image navigator is sampled using 10 ramp-up preparation pulses of the bSSFP readout with the same field-of-view (FOV) to the 3D acquisition [19]. The 2D iNAV is acquired in every heartbeat before the 3D imaging, allowing for foot-head (FH) and left-right (LR) translational respiratory motion estimation and correction, as described below. Example iNAV images acquired with different T1ρ preps are shown in Fig. 2a.

### Respiratory motion estimation and correction

The respiratory motion correction framework is shown in Fig. 2, which comprises two steps: 2D beat-to-beat translational motion correction for each T1ρ-weighted dataset separately and subsequent 3D translational alignment between the different T1ρ-weighted volumes (inter-contrast motion correction). For each T1ρ-weighted dataset, 2D FH and LR beat-to-beat translational respiratory motion is estimated from the iNAVs using a rectangular template that is manually selected around the heart on the first frame of iNAVs and propagated to all other frames (Fig. 2a). To find the reference end-expiration position, firstly, all iNAV frames are registered to the first frame, to obtain the FH motion curve, based on which the frames corresponding to end-expiration are selected by peak analysis of the FH motion. The average of FH motion at the selected end-expiration frames is calculated and the iNAV frame of which the FH motion is closest to the average is selected as the end-expiration reference frame. Then, all iNAV frames are translationally registered to the reference frame to obtain the FH and LR motion curves. Example of estimated FH motion curves for each T1ρ contrast are shown in Fig. 2b. The estimated beat-to-beat FH and LR translational shifts are used to correct the k-space phase of the corresponding spiral-like arm [19, 23]. Zero-filling reconstructions of the undersampled T1ρ-weighted datasets before and after 2D iNAV-based motion correction are shown in Fig. 2c and d, respectively. Beat-to-beat FH and LR motion compensated T1ρ-weighted datasets (Fig. 2d) are registered using mutual information metric to estimate remaining inter-contrast 3D translational motion with the volume of longest T1ρ prep (TSL = 50 ms) as reference. The 3D translational motion estimation was also focused on the heart region by selecting a rough rectangular box covering the whole heart. The estimated inter-contrast 3D translational motion parameters are used to correct the multi-contrast k-space dataset.

### Image reconstruction

After motion correction, the undersampled k-space was reconstructed using a multi-contrast 3D image patch-based low-rank algorithm (HD-PROST), which exploits local (image patch for a given pixel location), non-local (similar image patches searched in a surrounding region) and contrast redundancies of the 3D multi-contrast images [20]. The reconstruction is formulated as follows:

1 $$ {\displaystyle \begin{array}{c} argmin\\ {}I\end{array}}{\left\Vert MFCI-K\right\Vert}_F^2+\lambda {\sum}_p{\left\Vert {\mathcal{T}}_p\right\Vert}_{\ast },s.t.{\mathcal{T}}_p={P}_p(I) $$

where *I* are the multi-contrast 3D images to be reconstructed; *K* is the motion-corrected undersampled Cartesian k-space; *C* is the coil sensitivity maps which are estimated using the fully sampled k-space center region; *F* is 3D Fourier Transform; and *M* is the k-space sampling mask. *P*_*p*_(∙) selects similar 3D image patches for a given pixel location *p* from a 3D multi-contrast image set and forms a 3D tensor from the selected image patches. The selected image patches include similar patches around a given pixel location for all contrasts. ${\mathcal{T}}_p$ is a 3D tensor built by the selected patches centered at pixel *p*; ‖∙‖_*F*_ and ‖∙‖_∗_ denote the Frobenius norm and nuclear norm respectively; *λ* is the regularization parameter balancing the constraints of data fidelity and low-rankness. The reconstruction equation can be solved by operator-splitting via alternating direction method of multipliers (ADMM), as detailed in [20].

HD-PROST reconstruction parameters adopted in this study were empirically selected following the parameters suggested in [20] and by visual inspection of the reconstruction quality on one subject and used for all other datasets, which are: patch size = 5 × 5 × 5 pixels; search window radii around a pixel = 20 × 20 × 20; patch offset = 4; number of selected similar patches = 20; regularization parameter λ = 0.2; ADMM iterations = 5, leading to reconstruction time of ~ 25 mins. Example of multiple T1ρ-weighted images after HD-PROST reconstruction are shown in Fig. 2e.

### T1ρ mapping

T1ρ map is obtained by pixel-wise linear fitting of the logarithm of the T1ρ-weighted images. The signal equation for the saturation recovery T1ρ prepared acquisition is given by:

2 $$ S={S}_0\left({T}_{SR},{T}_1\right){\mathit{\exp}}^{- TSL/{T}_{1\rho }} $$

where *S* is the signal intensity of the given T1ρ-weighted image, *S*_0_(*T*_*SR*_, *T*_1_) is the signal before T1ρ prep, which is a saturation recovery function of *T*_*SR*_ and *T*_1_. After log transformation, the signal equation becomes:

3 $$ \ln \left(\mathrm{S}\right)=\ln \left({S}_0\left({T}_{SR},{T}_1\right)\right)-\frac{TSL}{T_{1\rho }} $$

The linear regression form of Eq. (3) can be written as *y* = *Ax* + *b*, with *y* being ln(S), *x* being *TSL* and the intercept *b* being ln(*S*_0_(*T*_*SR*_, *T*_1_)), the slope *A* being −1/*T*_1*ρ*_. Given different *x* and *y*, *A* and therefore T1ρ can be obtained by linear fitting.

### Phantom experiments

A phantom with different T1ρ values was imaged to test the accuracy of the proposed 3D T1ρ mapping using a 2D fully sampled T1ρ mapping acquisition as reference. The phantom consists of 6 vials containing gel and agarose in concentrations of 0.8, 1, 1.5, 2, 3, 5%, providing expected T1ρ values from 25 ms to 125 ms. The imaging settings for the 2D T1ρ mapping reference were: no SAT pulse; simulated heart rate of 20 bpm; every other heartbeat acquisition, resulting in magnetization recovery time of 6 s; a single k-space line was acquired after each T1ρ prep to eliminate the effect of T1 recovery during readout; 5 TSLs (0, 10, 20, 35, 50 ms); total scan time of 49.1 mins. Other imaging parameters for the 2D scan included: FOV = 288 × 200 mm^2^, spatial resolution = 2 × 2 mm^2^, slice thickness = 6 mm, TR/TE = 3.3/1.2 ms, flip angle = 50°..

Different heart rates lead to different trigger delays as the onset of the mid-diastolic quiescent phase is heart rate dependent, and thus result in different saturation recovery times. To investigate how heart rate influences T1ρ measurement, the 3D accelerated T1ρ mapping acquisition was performed with different simulated heart rates from 50 bpm to 110 bpm, with 10 bpm increment. The mid-diastolic trigger delays for different heart rates were calculated according to an empirical equation [24]. The 3D imaging parameters were the same as the in vivo imaging which are described in the next section.

### In vivo experiments

Eleven healthy subjects (five females, 30 ± 3 years) and three patients (one female, 59, 56, 26 years) with suspected cardiovascular disease were imaged. The study was approved by the institutional review board and all subjects gave written informed consent before imaging. All imaging experiments were performed on a 1.5 T scanner (MAGNETOM Aera; Siemens Healthineers, Erlangen, Germany) equipped with 18-channel body and 32-channel spine coils.

For healthy subjects, both the breath-hold 2D and the accelerated free-breathing 3D T1ρ mapping acquisitions were performed. The breath-hold 2D T1ρ mapping sequence was similar to the 3D sequence (Fig. 1a) including the SAT pulse to allow for data acquisition at every heartbeat. 2D T1ρ mapping was performed at the mid-ventricular short-axis location during mid-diastole within a single end-expiratory breath-hold for all five T1ρ contrasts to reduce cardiac motion and respiratory motion effects. 2D T1ρ mapping imaging parameters were: FOV = 300 × 300 mm^2^; spatial resolution = 2 × 2 mm^2^; slice thickness = 8 mm; TR/TE = 3.3/1.2 ms (partial Fourier in the readout direction); flip angle = 50° (balancing the trade-off between SNR and radiofrequency power deposition); five T1ρ prep spin-lock times, TSL = 0, 10, 20, 35, 50 ms; parallel imaging GRAPPA acceleration factor = 2; and 25 readouts per cardiac cycle with acquisition window < 100 ms to minimize cardiac motion [25]. Acquisition time was of 4 heart beats per contrast, resulting in a total breath-hold acquisition time of 20 heart beats for 2D T1ρ mapping. Free-breathing 3D T1ρ mapping was acquired during mid-diastole in coronal orientation with spatial resolution = 1.7 × 1.7 × 2 mm^3^, FOV = 300 × 300 mm^2^, TR/TE = 3.6/1.2 ms, flip angle = 50°, number of readouts per cardiac cycle ~ 35 (acquisition window ~ 120 ms to reduce cardiac motion) [25]. Total scan time for the proposed free-breathing 3D T1ρ mapping was 6.2 ± 0.6 mins (with a minimum of 5.4 mins and a maximum of 7.5 mins).

The proposed 3D free-breathing T1ρ images were acquired on all 3 patients before contrast injection. Approximately 15 min after contrast administration, ECG-gated LGE images were acquired to detect myocardial scar using a 2D phase-sensitive inversion recovery (PSIR) sequence under multiple breath-holds with spatial resolution = 1.4 × 1.4 × 8 mm^3^, inversion time = 145–160 ms to generate dark blood contrast [3] and flip angle = 45°. For patients, the breath-hold 2D T1ρ acquisition was not performed due to the long breath-hold time required (~20s).

### Image analysis

For the 2D and 3D phantom maps, a circular region-of-interest was drawn to cover the central region of each vial (containing ~ 112 pixels). Mean and standard variation (SD) of T1ρ values in the region-of-interest were calculated for each vial. The 3D T1ρ measurement accuracy and precision for different heart rates were calculated using the 2D T1ρ mapping as reference.

For mapping analysis of healthy subjects, the reconstructed 3D T1ρ map was reformatted to 3 short-axis (SAx) slices (base, mid and apex). The mean and SD of T1ρ were measured in the septum of the mid ventricular SAx map, which were compared with those of the 2D breath-hold scan using paired Wilcoxon rank-sum test. Bland-Altman analysis was performed to test the agreement of septal T1ρ measurements between the 3D and 2D methods for each subject. To evaluate the T1ρ distribution estimated by the proposed 3D T1ρ mapping, the 16-segment American Heart Association (AHA) analysis [26] was performed on the 3 reformatted SAx slices with 4 segments for apex, and 6 segments separately for mid and base. Mean T1ρ value in each segment was recorded and averaged across all heathy subjects and visualized using bullseye plot.

For patient data, the 3D T1ρ maps were reformatted to the slice positions as close as possible to that of the breath-hold 2D LGE images. Reformatting was performed using the physical coordinates of the 3D T1ρ and 2D LGE scans from the DICOM headers. However, perfect agreement cannot be ensured considering that the breath-hold position of 2D LGE and the end-expiration position considered in 3D T1ρ may be slightly different. The reformatted T1ρ maps were evaluated with the corresponding LGE PSIR images for scar detection.

## Results

### Phantom study

**Fig. 3:**
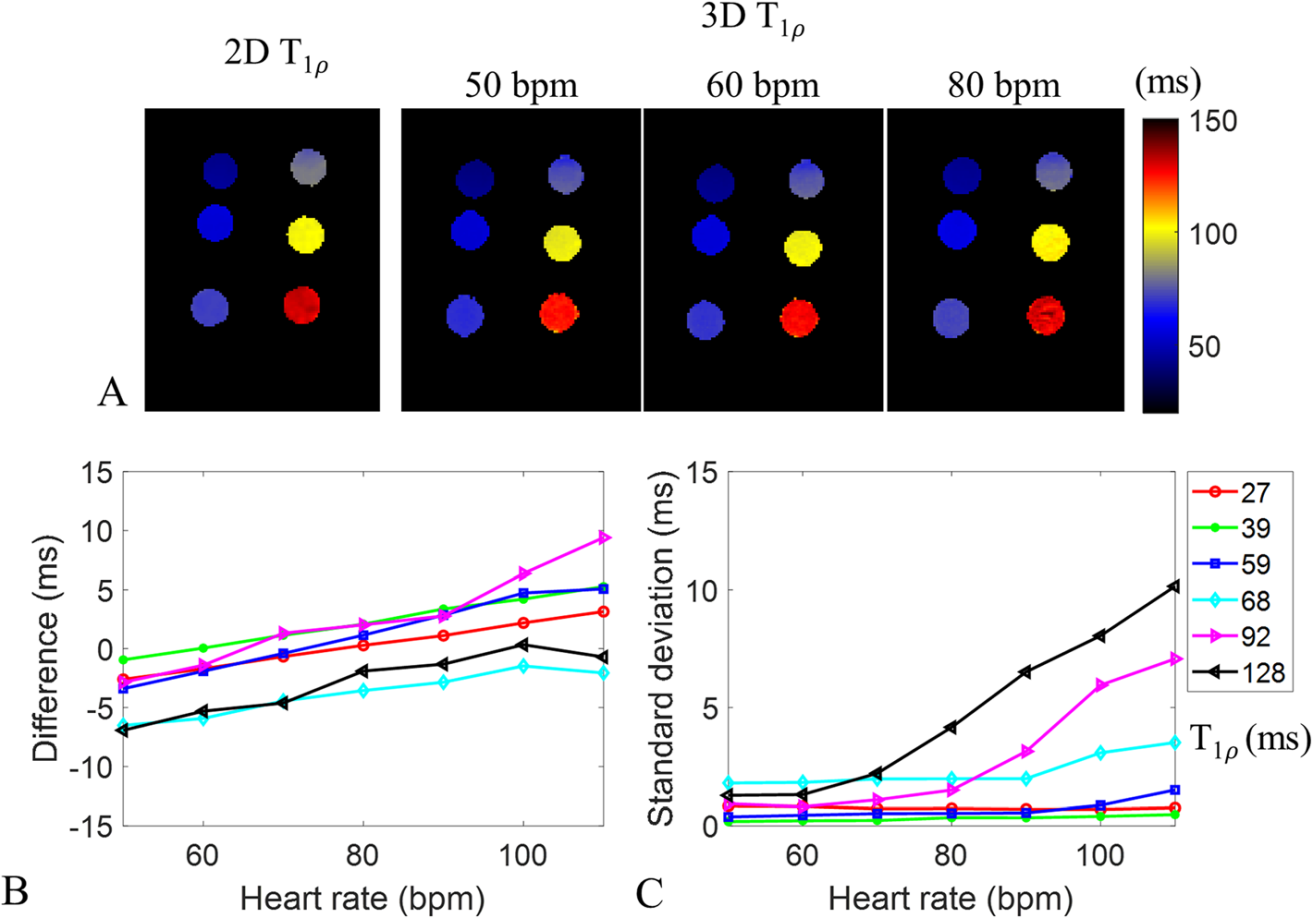
**a** Phantom T1ρ maps obtained with the 2D reference method and proposed 3D accelerated T1ρ mapping technique at simulated heart rates of 50, 60 and 80 bpm. **b** and **c** T1ρ measurement accuracy (difference between 3D and reference 2D measurements) and standard deviation of the 3D method with simulated heart rates of 50 to 110 bpm, in comparison with 2D reference

The phantom T1ρ maps estimated by the 2D method and the proposed 3D approach with simulated heart rates of 50, 60 and 80 bpm are shown in Fig. 3a. In comparison with 2D T1ρ mapping, the measurement accuracy and precision (measured as SD) of 3D T1ρ mapping for different heart rates are provided in Fig. 3b, c. Similar accuracy was observed for a wide range of simulated heart rates, while measurement precision of longer T1ρ values decreased with higher heart rates.

### In vivo study

**Fig. 4:**
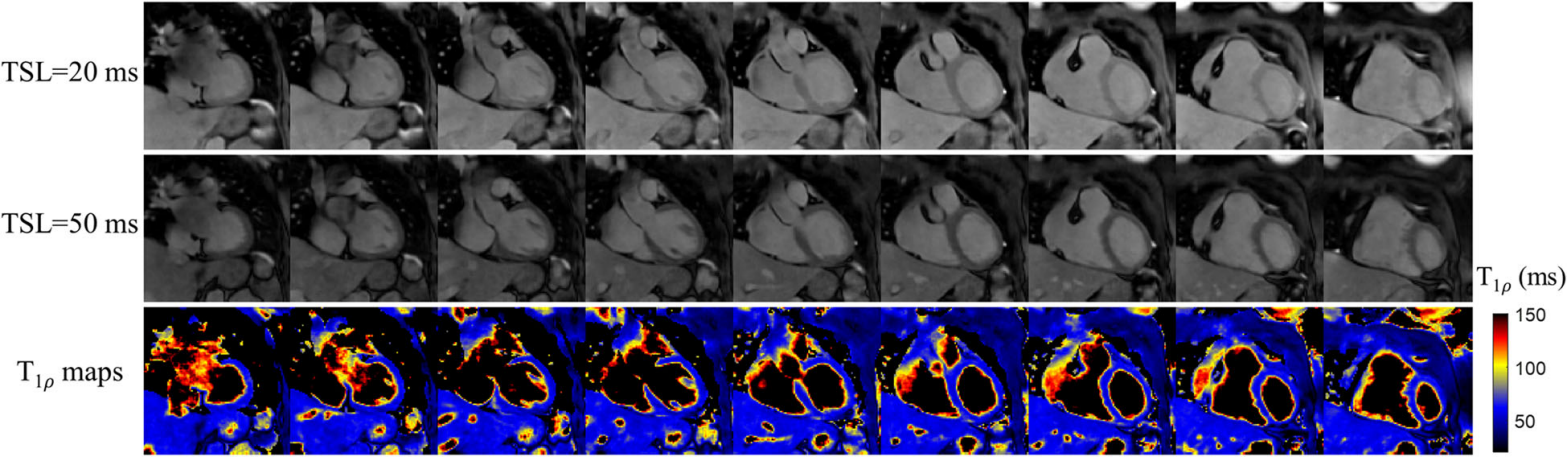
3D T1ρ mapping for a healthy subject (heart rate: 76 bpm; acquisition duration: 5.4 min) with the proposed approach. Nine acquired coronal slices are shown for representative T1ρ-weighted images of spin-lock time (TSL) of 20 and 50 ms in the first two rows, and the fitted T1ρ maps at the bottom

**Fig. 5:**
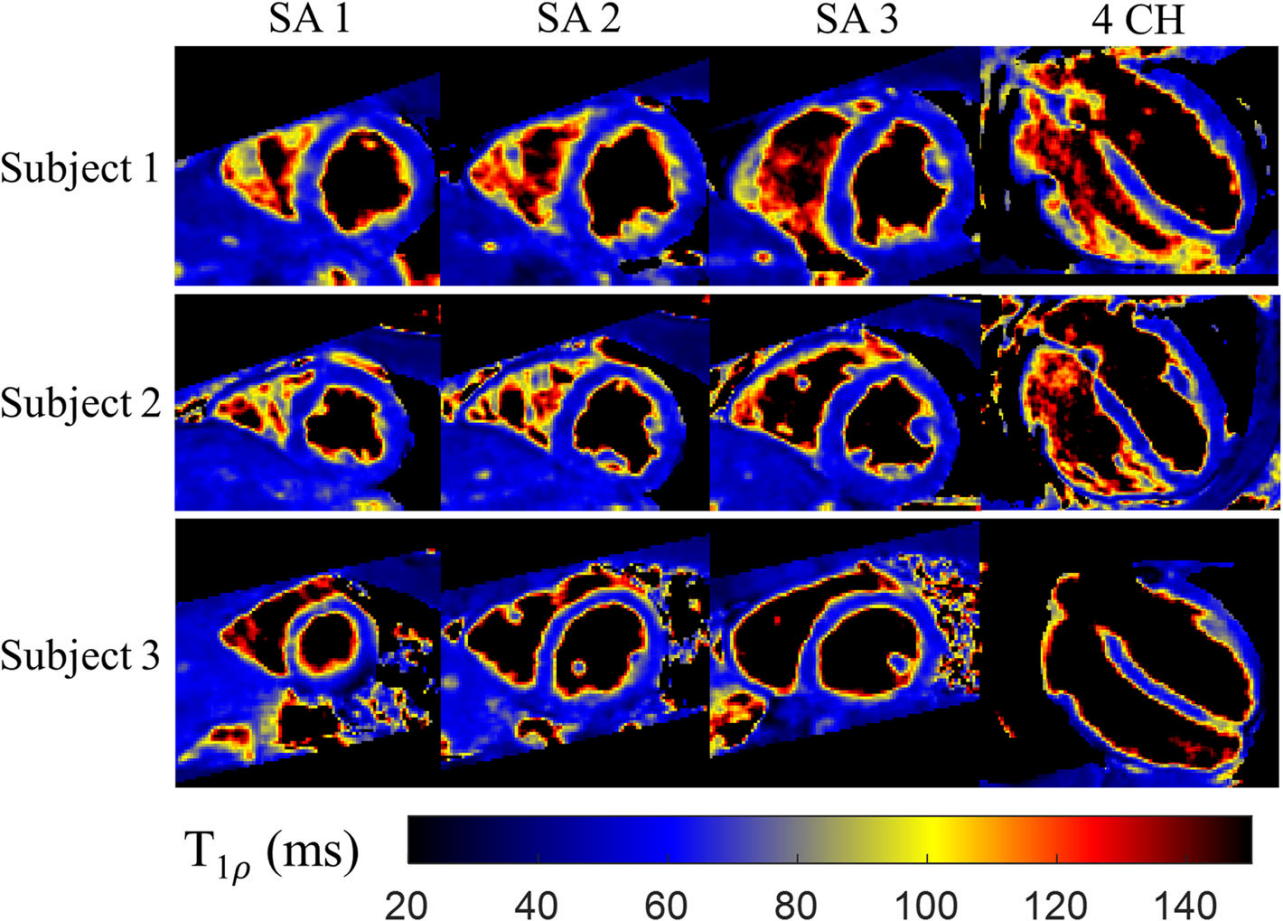
Reformatted 3D T1ρ maps from 3 healthy subjects (heart rates: 68, 54, 72 bpm; acquisition durations: 6.1, 6.3, 5.4 min), including 3 short-axis (SAx) slices and the long-axis 4 chamber (4 CH) view

T1ρ maps were successfully reconstructed for all 14 subjects. Representative T1ρ-weighted images and T1ρ maps of one healthy subject with the proposed 3D free-breathing approach are shown in Fig. 4, including 9 coronal slices. Good quality T1ρ-weighted images, as well as T1ρ maps were obtained with the proposed framework. Reformatted T1ρ maps of the apical, mid, basal SA and 4-chamber (4 CH) views from three additional healthy subjects are shown in Fig. 5.

**Fig. 6:**
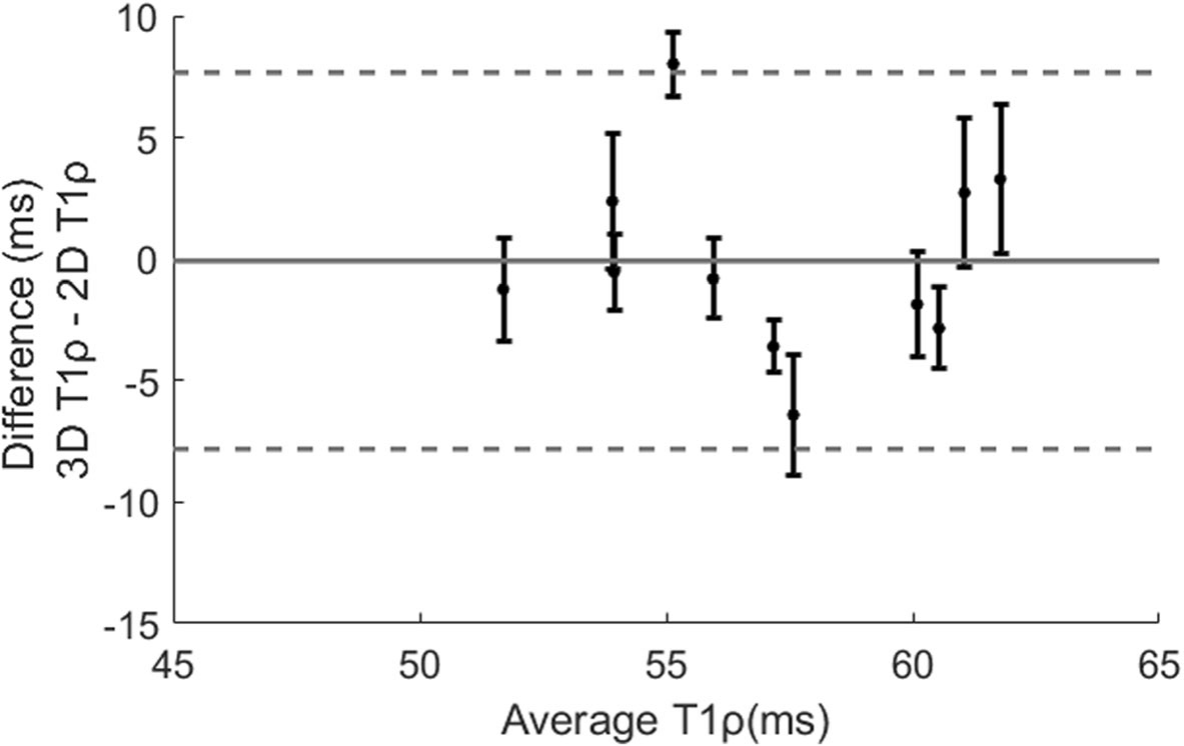
Bland-Altman analysis of the septal T1ρ values (mid SAx slice) of 11 healthy subjects measured with breath-hold 2D T1ρ and free-breathing 3D T1ρ mapping methods. The black dot shows the bias and the bars show the 95% confidence intervals of the bias for each subject. The grey solid line indicates the mean difference across all subjects, and the grey dotted lines indicate the 95% confidence intervals of population bias

**Fig. 7:**
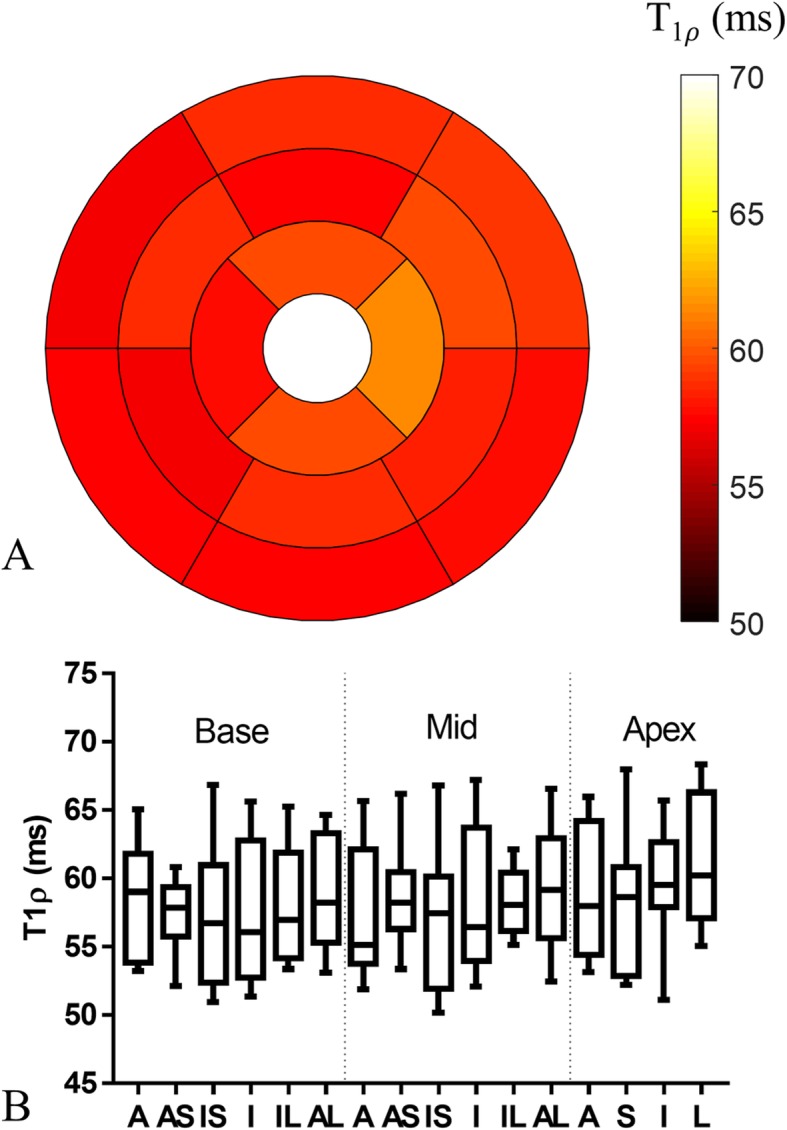
**a** American Heart Association (AHA) bullseye plot showing the myocardial T1ρ distribution across the left ventricle measured with the proposed free-breathing 3D T1ρ mapping technique. **b** Box plot showing the median, 25 and 75 percentiles, and range of T1ρ values in each AHA segment. (A: anterior; AS: anteroseptal; IS: inferoseptal; I: inferior; IL: inferolateral; AL: anterolateral; S: septal; L: lateral)

Septal T1ρ values of the 11 healthy subjects measured with the proposed 3D free-breathing approach were 58.0 ± 4.1 ms, in close agreement with values measured with the breath-hold 2D T1ρ (57.6 ± 4.7 ms, *P* = 0.83). Bland-Altman analysis of the septal T1ρ measurements is demonstrated in Fig. 6, where the 95% confidence interval of the bias is shown for each subject and the whole population. Bland-Altman analysis indicated a good agreement (mean population bias − 0.1 ms) between the 3D and 2D methods. The 3D myocardial T1ρ maps of healthy subjects showed uniform distribution. The T1ρ of each AHA segment is shown in the bullseye and box plots in Fig. 7.

**Fig. 8:**
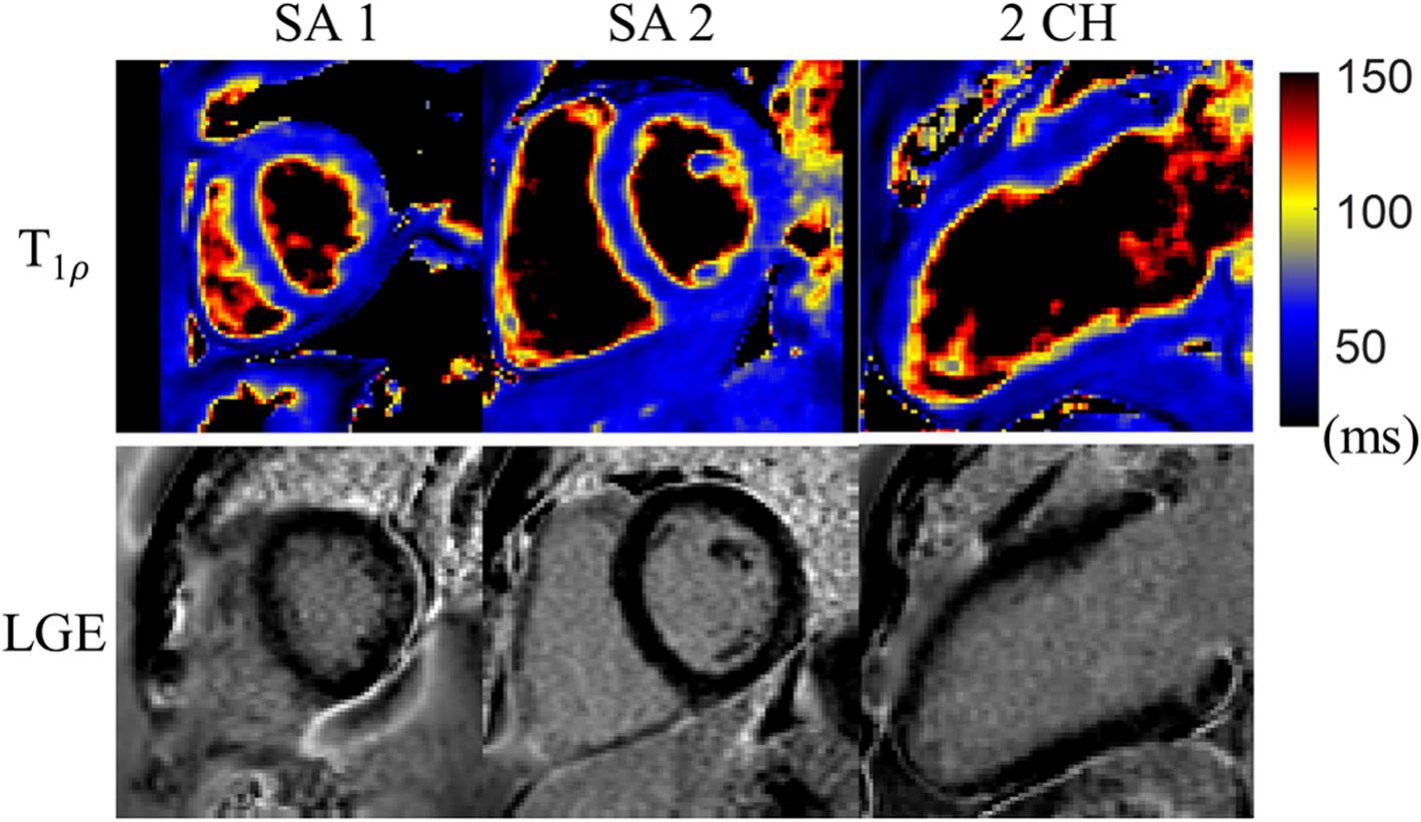
Reformatted 3D T1ρ maps and 2D LGE images of a patient (heart rate: 63 bpm; acquisition duration: 6.2 min) without myocardial infarction, including two short-axis (SAx) slices and the long-axis 2 chamber (2 CH) view

**Fig. 9:**
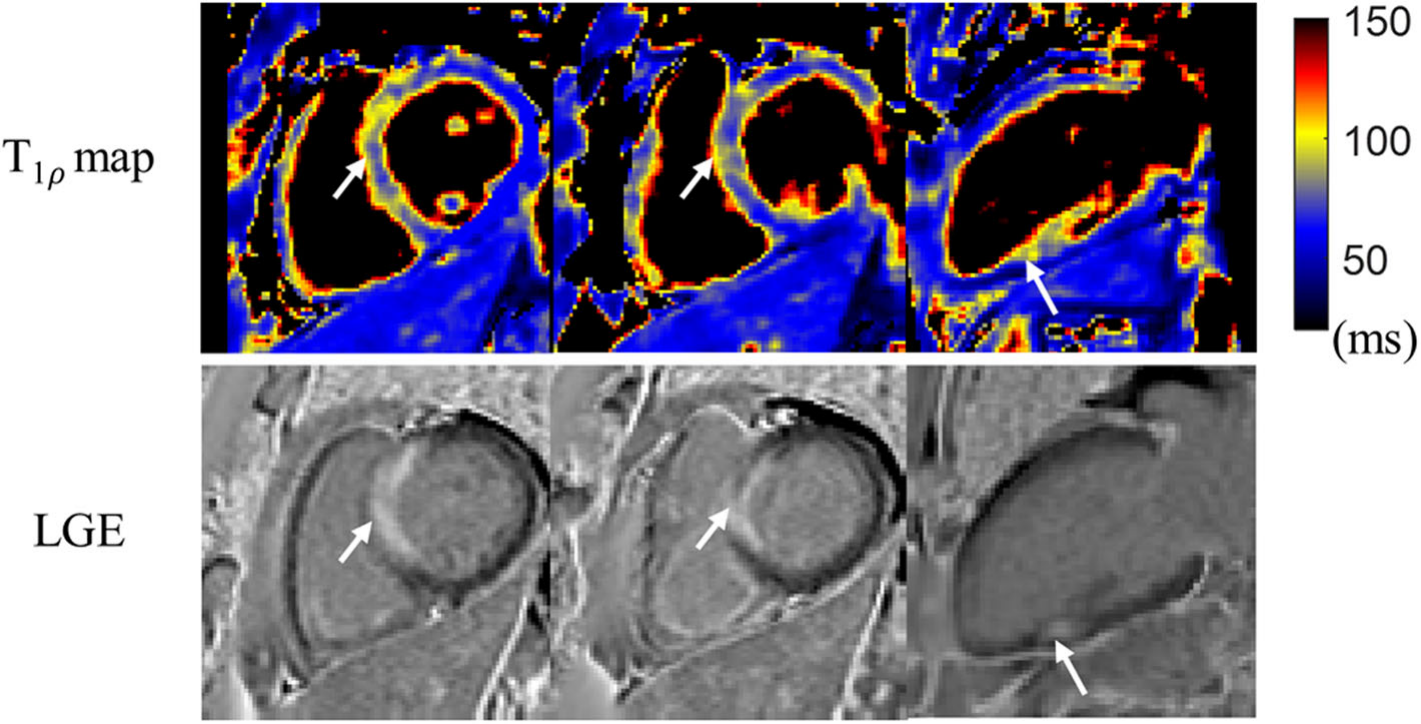
Reformatted 3D T1ρ maps and 2D LGE images of a patient (heart rate: 69 bpm; acquisition duration: 5.8 min) with myocardial infarction. The white arrows indicate the scar regions on the short-axis (SAx) and 2 chamber (2 CH) views, as detected by enhancement on LGE images. Increased values in T1ρ maps were observed for the infarcted regions

According to the clinical 2D LGE images, scar was detected in one of the three patients. Reformatted T1ρ maps from the proposed 3D free-breathing scan of one patient without myocardial infarction are shown along with the LGE images in Fig. 8. No myocardial enhancement was observed on LGE and no alteration was observed on the T1ρ maps. The scar patient results are demonstrated in Fig. 9, including the LGE images and reformatted 3D T1ρ maps in SAx and 2 CH views. The infarcted myocardium showed hyperintensity on LGE, and higher T1ρ values (87.4 ± 5.7 ms) than the healthy myocardium (61.9 ± 2.2 ms) were observed in the scar region with the proposed 3D T1ρ mapping technique.

## Discussion

In this study, a free-breathing 3D T1ρ mapping technique was proposed featuring whole heart coverage, near-isotropic spatial resolution (1.7 × 1.7 × 2 mm^3^) and 100% respiratory acquisition efficiency. Combining the efficient respiratory acquisition, the adoption of saturation pulse to avoid recovery heartbeats and 3.8-fold undersampled VD-CASPR sampling [18], five differently T1ρ-weighted volumes were acquired in a clinically feasible scan time (~ 6 mins), based on which 3D T1ρ maps were estimated. The accuracy of the 3D technique was investigated in phantoms and healthy subjects, while clinical feasibility was explored in 3 patients.

The achievable spatial resolution and coverage are limited with 2D T1ρ mapping, due to breath-holding constraint. Multiple breath-holds may lead to mapping inaccuracy due to inconsistency in respiratory positions and are challenging in some patient populations. Respiratory gating by diaphragmatic navigator has recently been used to enable free-breathing 3D T1ρ mapping [16]. However, diaphragmatic navigator gating may result in long and unpredictable scan times due to the low acquisition efficiency. The use of a 2D image-based navigator enables free-breathing scan with 100% respiratory acquisition efficiency, making near-isotropic whole heart T1ρ maps feasible in a predictable and clinically acceptable scan time.

In the ECG-triggered acquisition, the predominant difference of heart position is in the FH direction induced by respiration [27]. To minimize respiratory motion artifacts, coronal 2D iNAVs were used to estimate FH and LR translational motion to correct the corresponding k-space phase in each heartbeat. 2D iNAV-based motion estimation / correction was performed for each T1ρ contrast separately, to avoid influence of the contrast differences on the motion estimation. Subsequently, inter-contrast 3D translational motion correction was performed to align all the T1ρ-weighted images in case the end-expiration reference positions are different between T1ρ contrasts. Translational motion estimation and correction is fast and robust, and has been found to efficiently reduce respiration motion artifacts in CMR [28, 29]. Thus, it was used in this study to correct respiratory motion. However, affine or non-rigid motion correction could be incorporated in future studies to correct for remaining respiratory motion.

ECG-triggered 3D multi-contrast acquisitions for mapping remain lengthy in spite of the use of more efficient respiratory motion correction with iNAVs as described above. Feasibility of accelerated 3D myocardial T1ρ mapping using compressed sensing with spatial total variation regularization has been demonstrated in a recent study [16]. This approach used the same undersampling pattern for all contrasts and did not exploit redundancies in the parameter (contrast) dimension. In this study, a variable-density Cartesian undersampling with spiral-like ordering in the ky-kz plane was adopted [18]. The spiral-like arms acquired in each cardiac cycle are shifted with golden-angle step within each contrast to introduce incoherent undersampling artifacts. Additionally, a golden-angle shift was also employed between T1ρ contrasts so that artifacts are also incoherent in the contrast dimension and an additional sparsity constraint can thus be effectively imposed. HD-PROST reconstruction which exploits local (within a patch), non-local (between neighbouring patches) and contrast (along the T1ρ parameter dimension) redundancies [20] was employed to efficiently reduce undersampling artifacts.

Acceleration factor of 3.8 was adopted in this study to achieve clinically feasible scan times (~ 6mins). To explore the feasibility of further acceleration, the dataset acquired with acceleration factor of 3.8 was retrospectively undersampled to a factor of 5x. The reconstructed images and T1ρ maps are provided in Additional file 1: Figure S1. Acceleration factor of 5 generally achieved acceptable performance with slight loss of image and T1ρ mapping quality compared with acceleration factor of 3.8. Further acceleration of the 3D T1ρ mapping technique to facilitate faster scanning or higher spatial resolution may be feasible, but optimization of the reconstruction process would be required.

T1ρ techniques have shown promising results in a variety of applications such as knee cartilage, liver and brain [9–13]. However, conventional T1ρ prep with spin-lock pulse of constant amplitude is known to be sensitive to B1 and B0 field inhomogeneities [30, 31]. Several improvements have been proposed. The rotary echo method was proposed to remove banding artifacts due to B1 inhomogeneity [32] with however the influence of B0 inhomogeneity unsolved. By combining rotary echo with a 180° refocusing pulse, the radiofrequency pulse cluster can compensate for B0 imperfections, but it is sensitive to B1 inhomogeneity [30]. In this study, two 180° refocusing pulses with opposite phases were introduced to generate a balanced radiofrequency pulse cluster and make the T1ρ prep more robust to B1 and B0 inhomogeneities [21, 22]. T1ρ prep with a chain of adiabatic pulses is another approach that has been shown to be insensitive to B1 and B0 inhomogeneities [33]. However, the time-varying amplitude spin-lock by adiabatic pulses and the constant amplitude spin-lock reflects different tissue properties [33]. Future studies will be performed to compare these two kinds of T1ρ prep for cardiac imaging applications.

In conventional myocardial T1ρ mapping, data is acquired every other heartbeat to ensure restoration of the magnetization before T1ρ preparation [15, 16]. However, with this approach, besides increasing the scan time, heartrate variations during the scan will result in variable T1 recovery times and therefore influence T1ρ estimation. In the proposed sequence, a saturation pulse was introduced to reset the magnetization at the start of each cardiac cycle, similar to the 3D cardiac T2 mapping study by Ding et al. [17], to enable efficient data acquisition at every heartbeat. The saturation pulse with constant saturation recovery time (the time between SAT and T1ρ prep) provides the same magnetization before each T1ρ preparation, making the sequence insensitive to heart rate variability during acquisition [17].

In phantoms, the proposed 3D accelerated T1ρ mapping technique achieved good accuracy for a wide range of simulated heart rates. However, the shorter saturation recovery time for higher heart rate will lead to lower SNR of the acquired T1ρ-weighted images, which lead to a decrease of measurement precision for longer T1ρ values (92 ms and 128 ms). The T1ρ decay range for shorter T1ρ is larger than longer T1ρ, so the measurement precision of shorter T1ρ values is less affected by heart rates (T_SR_) than that of longer ones. One option to improve the performance for longer T1ρ values is to increase the spin-lock duration, which, however, will increase the specific absorption rate and requires more powerful radiofrequency amplifier. For septal T1ρ measurement in healthy subjects, a good agreement was observed between the proposed free-breathing 3D and breath-hold 2D T1ρ mapping methods. Homogeneous 3D T1ρ maps were obtained through the whole left ventricle in healthy subjects, with minor fluctuations observed between segments in the AHA analysis. In particular, the apex segments showed higher T1ρ values, which may be attributed to residual motion effects.

For the patient with myocardial infarction according to LGE images, higher T1ρ values were observed for the scar region in comparison to the remote normal myocardium. Previous imaging studies of ex-vivo and swine model of myocardial infarction [7, 8] also reported increased T1ρ values in infarcts. The mechanism causing higher T1ρ is still not fully understood, as there are several factors that may influence T1ρ signal, such as water molecular motion, molecular diffusion, chemical exchange and magnetization transfer [8]. Witschey et al. [7] suggest that increased T1ρ may be attributed to increased water mobility in scar which leads to shorter nuclear rotational correlation times and longer transverse relaxation time.

There are several limitations to this study. First, spin-lock amplitude of 400 Hz was used for T1ρ prep. The spin-lock amplitude in this study was limited by the power capacity of the radiofrequency amplifier of the available clinical scanner. T1ρ times increase with spin-lock frequency and higher T1ρ values should be expected if higher spin-lock amplitude can be employed. If hardware permits, larger spin-lock amplitude should be used as previous study suggested higher T1ρ sensitivity at higher spin-lock amplitudes [8]. Second, there is no data rejection for arrhythmia in the current sequence implementation. Arrhythmia is a common issue for ECG-triggered acquisitions. Arrhythmia rejection will be investigated to minimize cardiac motion artifacts in future clinical studies. Third, a limited number of patients was included in this study to show preliminary feasibility of the proposed framework. Validation of the proposed approach in a larger number of patients with ischemic cardiac disease is warranted to investigate the sensitivity of the proposed 3D T1ρ mapping for myocardial infarction detection.

## Conclusions

In this study, a novel free-breathing 3D whole heart T1ρ mapping technique was proposed, which enabled 100% respiratory scan efficiency and near-isotropic resolution (1.7 × 1.7 × 2 mm^3^) in a clinically feasible scan time of ~ 6 mins, achieving similar accuracy to breath-hold 2D T1ρ mapping. Preliminary patient results suggest that the proposed technique may find applications in non-contrast myocardial tissue characterization.

## Supplementary information

**Additional file 1: Figure S1.** Reconstructed T1ρ image and T1ρ map of a heathy subject with undersampling factors of 3.8 and 5. The 5x accelerated data was obtained by retrospective undersampling of the data acquired with acceleration factor 3.8. Slight loss of reconstruction and mapping quality can be observed for undersampling factor 5, indicated by the white arrows.

## Data Availability

The datasets used and/or analysed during the current study are available from the corresponding author on reasonable request.

## Abbreviations

2 CH: 2-chamber
4 CH: 4-chamber
ADMM: Alternating direction method of multipliers
AHA: American Heart Association
bSSFP: Balanced steady-state free-precession
CMR: Cardiovascular magnetic resonance
ECG: Electrocardiogram
FH: Foot-head
FOV: Field of view
HD-PROST: Multi-contrast 3D patch-based low-rank reconstruction
iNAV: Image based navigator
LGE: Late gadolinium enhancement
LR: Left-right
PSIR: Phase-sensitive inversion recovery
SAT: Saturation pulse
SAx: Short-axis
SD: standard variation
SNR: Signal-to-noise ratio
T1ρ prep: T1ρ preparation
TSL: Spin lock time
VD-CASPR: Variable-density 3D Cartesian sampling with spiral-like order

## References

[1] Sutton MGS, Sharpe N (2000). Left ventricular remodeling after myocardial infarction - pathophysiology and therapy. Circulation.

[2] Holtackers RJ, Van De Heyning CM, Nazir MS, Rashid I, Ntalas I, Rahman H (2019). Clinical value of dark-blood late gadolinium enhancement cardiovascular magnetic resonance without additional magnetization preparation. J Cardiovasc Magn Reson.

[3] Holtackers RJ, Chiribiri A, Schneider T, Higgins DM, Botnar RM (2017). Dark-blood late gadolinium enhancement without additional magnetization preparation. J Cardiovasc Magn Reson.

[4] Ledneva E, Karie S, Launay-Vacher V, Janus N, Deray G (2009). Renal safety of gadolinium-based contrast Media in Patients with chronic renal insufficiency. Radiology.

[5] Kanda T, Ishii K, Kawaguchi H, Kitajima K, Takenaka D (2014). High signal intensity in the dentate nucleus and Globus Pallidus on unenhanced T1-weighted MR images: relationship with increasing cumulative dose of a gadolinium-based contrast material. Radiology.

[6] Wang L, Yuan J, Zhang SJ, Gao M, Wang YC, Wang YX (2016). Myocardial T1rho mapping of patients with end-stage renal disease and its comparison with T1 mapping and T2 mapping: a feasibility and reproducibility study. J Magn Reson Imaging.

[7] Witschey WRT, Zsido GA, Koomalsingh K, Kondo N, Minakawa M, Shuto T (2012). In vivo chronic myocardial infarction characterization by spin locked cardiovascular magnetic resonance. J Cardiovasc Magn Reson.

[8] Han Y, Liimatainen T, Gorman RC, Witschey WR (2014). Assessing myocardial disease using T1rho MRI. Curr Cardiovasc Imaging Rep.

[9] Witschey WRT, Borthakur A, Fenty M, Kneeland BJ, Lonner JH, McArdle EL (2010). T1 rho MRI quantification of arthroscopically confirmed cartilage degeneration. Magn Reson Med.

[10] Wang LG, Chang G, Xu J, Vieira RLR, Krasnokutsky S, Abramson S (2012). T1rho MRI of menisci and cartilage in patients with osteoarthritis at 3T. Eur J Radiol.

[11] Chen WB, Chen X, Yang L, Wang GB, Li JQ, Wang SS (2018). Quantitative assessment of liver function with whole-liver T1rho mapping at 3.0 T. Magn Reson Imaging.

[12] Allkemper T, Sagmeister F, Cicinnati V, Beckebaum S, Kooijman H, Kanthak C (2014). Evaluation of fibrotic liver disease with whole-liver T1 rho MR imaging: a feasibility study at 1.5 T. Radiology.

[13] Grohn OHJ, Kettunen MI, Makela HI, Penttonen M, Pitkanen A, Lukkarinen JA (2000). Early detection of irreversible cerebral ischemia in the rat using dispersion of the magnetic resonance imaging relaxation time, T-1p. J Cerebr Blood F Met.

[14] Muthupillai R, Flamm SD, Wilson JM, Pettigrew RI, Dixon WT (2004). Acute myocardial infarction: tissue characterization with T1(rho)-weighted MR imaging - initial experience. Radiology.

[15] Berisha S, Han J, Shahid M, Han YC, Witschey WRT (2016). Measurement of Myocardial T-1 rho with a motion corrected, parametric mapping sequence in humans. PLoS One.

[16] Iyer SK, Moon B, Hwuang E, Han YC, Solomon M, Litt H (2019). Accelerated free-breathing 3D T1 cardiovascular magnetic resonance using multicoil compressed sensing. J Cardiovasc Magn Reson.

[17] Ding HY, Fernandez-De-Manuel L, Schar M, Schuleri KH, Halperin H, He L (2015). Three-dimensional whole-heart T2 mapping at 3T. Magn Reson Med.

[18] Prieto C, Doneva M, Usman M, Henningsson M, Greil G, Schaeffter T (2015). Highly efficient respiratory motion compensated free-breathing coronary MRA using Golden-step Cartesian acquisition. J Magn Reson Imaging.

[19] Henningsson M, Koken P, Stehning C, Razavi R, Prieto C, Botnar RM (2012). Whole-heart coronary MR angiography with 2D self-navigated image reconstruction. Magn Reson Med.

[20] Bustin A, Lima da Cruz G, Jaubert O, Lopez K, Botnar RM, Prieto C (2019). High-dimensionality undersampled patch-based reconstruction (HD-PROST) for accelerated multi-contrast MRI. Magn Reson Med.

[21] Gram M, Gensler D, Xu A, Nordbeck P, Bauer WR, Jakob PM (2019). A totally balanced spin lock preparation module for accurate and artifact-free T1ρ-mapping.

[22] Guo R, Si D, Luo J, Ding HY (2019). Two-dimensional simultaneous myocardial T1 and T1rho Mapping at 3T.

[23] Aitken AP, Henningsson M, Botnar RM, Schaeffter T, Prieto C (2015). 100% efficient three-dimensional coronary MR angiography with two-dimensional beat-to-beat translational and bin-to-bin affine motion correction. Magn Reson Med.

[24] Roes SD, Korosoglou G, Schar M, Westenberg JJ, van Osch MJP, de Roos A (2008). Correction for heart rate variability during 3D whole heart MR coronary angiography. J Magn Reson Imaging.

[25] Johnson KR, Patel SJ, Whigham A, Hakim A, Pettigrew RI, Oshinski JN (2004). Three-dimensional, time-resolved motion of the coronary arteries. J Cardiovasc Magn Reson.

[26] Cerqueira MD, Weissman NJ, Dilsizian V, Jacobs AK, Kaul S, Laskey WK (2002). Standardized myocardial segmentation and nomenclature for tomographic imaging of the heart: a statement for healthcare professionals from the Cardiac Imaging Committee of the Council on Clinical Cardiology of the American Heart Association. J Am Soc Echocardiog.

[27] Scott AD, Keegan J, Firmin DN (2009). Motion in cardiovascular MR imaging. Radiology.

[28] Manke D, Nehrke K, Bornert P, Rosch P, Dossel O (2002). Respiratory motion in coronary magnetic resonance angiography: a comparison of different motion models. J Magn Reson Imaging.

[29] Bustin A, Ginami G, Cruz G, Correia T, Ismail TF, Rashid I (2019). Five-minute whole-heart coronary MRA with sub-millimeter isotropic resolution, 100% respiratory scan efficiency, and 3D-PROST reconstruction. Magn Reson Med.

[30] Witschey WR, Borthakur A, Elliott MA, Mellon E, Niyogi S, Wallman DJ (2007). Artifacts in T1 rho-weighted imaging: compensation for B(1) and B(0) field imperfections. J Magn Reson.

[31] Chen W (2015). Errors in quantitative T1rho imaging and the correction methods. Quant Imaging Med Surg.

[32] Charagundla SR, Borthakur A, Leigh JS, Reddy R (2003). Artifacts in T-1 rho-weighted imaging: correction with a self-compensating spin-locking pulse. J Magn Reson.

[33] Mangia S, Liimatainen T, Garwood M, Michaeli S (2009). Rotating frame relaxation during adiabatic pulses vs. conventional spin lock: simulations and experimental results at 4 T. Magn Reson Imaging.

