# Supplementary material for: Respiratory motion-compensated high-resolution 3D whole-heart T1ρ mapping
Source: J Cardiovasc Magn Reson. 2020 Feb 3;22:12. doi: 10.1186/s12968-020-0597-5 (PMC6998259; doi:10.1186/s12968-020-0597-5)
Supplement: Supplementary file 1 — Additional file 1: Figure S1. Reconstructed T1ρ image and T1ρ map of a heathy subject with undersampling factors of 3.8 and 5. The 5x accelerated data was obtained by retrospective undersampling of the data acquired with acceleration factor 3.8. Slight loss of reconstruction and mapping quality can be observed for undersampling factor 5, indicated by the white arrows. [file 12968_2020_597_MOESM1_ESM.docx]

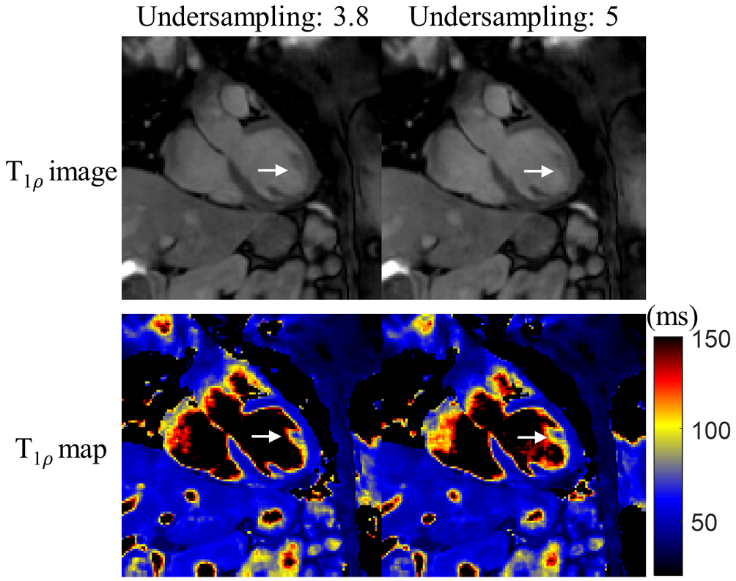


Figure S1: Reconstructed T1ρ image and T1ρ map of a heathy subject with undersampling factors of 3.8 and 5. The 5x accelerated data was obtained by retrospective undersampling of the data acquired with acceleration factor 3.8. Slight loss of reconstruction and mapping quality can be observed for undersampling factor 5, indicated by the white arrows.
